# Polynomially Quasi-*mth*-Helton class of Hilbert spaces operators

**DOI:** 10.1371/journal.pone.0355301

**Published:** 2026-09-02

**Authors:** Asma Al Rwaily

**Affiliations:** Department of Mathematics, College of Science, Qassim University, Buraydah, Saudi Arabia; Piri Reis University: Piri Reis Universitesi, TÜRKIYE

## Abstract

In this paper, we introduce and investigate a new class of bounded linear operators on a Hilbert space, called the *polynomially quasi-mth Helton class of operators* of order m∈ℕ. This class is defined through a vanishing operator relation involving a polynomial perturbation of the Helton operator map, namely.

$$\begin{array}{c} 𝒫(𝒜{)}^{*}\Phi (ℬ;𝒜{)}^{m}(ℐ)𝒫(𝒜)=0, \end{array}$$

for some nonconstant polynomial 𝒫, where Φ(ℬ;𝒜) denotes the Helton operator map given by

$$\begin{array}{c} \Phi (ℬ;𝒜)(X)=ℬX-X𝒜, \end{array}$$

so that

Φ(ℬ;𝒜)m(ℐ)=∑j=0m(−1)j(mj)ℬj𝒜m−j.

This framework extends the classical Helton class and the quasi-Helton class of order *m* by incorporating polynomial operator relations. We establish several fundamental properties of this class and investigate its structural behavior. In particular, we obtain characterizations, study stability properties, and discuss connections with well-known operator classes such as *m*-isometric and *m*-symmetric operators. Our results provide a unified framework that highlights the interplay between polynomial operator expressions and quasi-Helton-type relations in operator theory.

## 1. Introduction

In this paper, let 𝒦 denote an infinite-dimensional complex Hilbert space. We denote by ℒ(𝒦) the Banach algebra consisting of all bounded linear operators on 𝒦. For any operator A∈ℒ(K), we write ran(A) for its range, ker(A) for its kernel, and $A^{*}$ for its adjoint. Let 𝒜∈L(K), and define


Λl(𝒜,𝒜*):=∑0≤k≤l(−1)l−k(lk)𝒜*k𝒜k. (1)

It follows directly from (1) that


$$\begin{array}{c} \Lambda_{l+1}(𝒜,{𝒜}^{*})={𝒜}^{*}\Lambda_{l}(𝒜,{𝒜}^{*})𝒜-\Lambda_{l}(𝒜,{𝒜}^{*}). \end{array}$$ (2)

A Hilbert space operator 𝒜∈L(𝒦) is said to be an *m*-isometry ([1–3]), if 𝒜 satisfies the following operator equation


∑0≤k≤m(−1)m−k(mk)𝒜*k𝒜k=0, (3)

and it is said to be an *n*-quasi-*m*-isometry ([4,5]) if


𝒜*nΛm(𝒜,𝒜*)𝒜n=𝒜*n(∑0≤k≤m(−1)m−k(mk)𝒜*k𝒜k)𝒜n=0, (4)

for some integer m≥1. A comprehensive study of these classes of Hilbert space operators can be found in [1–3,5,6] and among other references. In [7], J.W. Helton introduced *m* -symmetric operators as a tool for the study of Jordan operators. An operator 𝒜∈ℒ(𝒦) is said to be *n*-symmetric or equivalently, an *n*-symmetry for some positive integer *n* if 𝒜 satisfies ([8,9])


γn(𝒜,𝒜*):=∑0≤k≤n(−1)n−k(nk)𝒜*k𝒜n−k=0. (5)

Then it holds


$$\begin{array}{c} \gamma_{n+1}(𝒜,{𝒜}^{*})={𝒜}^{*}\gamma_{n}(𝒜,{𝒜}^{*})-\gamma_{n}(𝒜,{𝒜}^{*})𝒜. \end{array}$$ (6)

Using (6), if 𝒜 is *n*-symmetric, then 𝒜 is (*n* + *k*)-symmetric for each integer k≥1 ([28, Proposition 28]). Investigations into the sum and product of *n*-symmetric operators were carried out in [10] and [9].

Let 𝒜 and ℬ be in ℒ(𝒦). In [11], the authors studied the operator Φ(𝒜,ℬ):ℒ(𝒦)⟶L(𝒦) defined by Φ(ℬ;𝒜)(X)=𝒜X−Xℬ. Then


Φ(ℬ;𝒜)k(ℐ)=∑0≤j≤k(−1)k−j(kj)ℬj𝒜k−j. (7)

The class of Helton operators was introduced by the authors in [11] as follows: an operator 𝒜∈ℒ(K) is said to be in the *mth* Helton class of ℬ∈ℒ(𝒦) and write $𝒜\in [{HEL}_{m}(ℬ)]$ if


$$\begin{array}{c} \Phi (ℬ;𝒜{)}^{m}(ℐ)=0. \end{array}$$ (8)

For more details, interested readers are referred to ([12,13]).

In a recent work [14], the author introduced the class of *n*-quasi-Helton operators of order

*m* as follows. An operator ℬ∈L(K) for which there exists an 𝒜∈L(K) and there are some integers *n* and *m* such that


𝒜*nΦ(ℬ;𝒜)m(ℐ)𝒜n=𝒜*n(∑0≤j≤m(−1)j(mj)ℬj𝒜m−j)𝒜n=0, (9)

According to the author, 𝒜 is a member of the *n*-quasi-Helton class associated to ℬ with order *m*. This is expressed symbolically as $𝒜\in [n𝒬]\cap [{HEL}_{m}(ℬ)]$.

Several authors have extended the notion of normal operators to that of polynomially normal operators and *n*-quasi-*m*-isometry to polynomially quasi-*m*-isometry. An operator𝒜∈L(K) is called polynomially normal if there exists a nonconstant complex polynomial 𝒫 such that


$$\begin{array}{c} 𝒫(𝒜){𝒜}^{*}-{𝒜}^{*}𝒫(𝒜)=0 \end{array}$$ (10)

([15–17]). Following a similar approach,an operator 𝒜∈L(K) is called polynomially quasi *m*-isometry ([18]) if there exists a nonconstant complex polynomial 𝒫 such that


𝒫(𝒜)*(∑0≤k≤m(−1)m−k(mk)𝒜*k𝒜k)𝒫(𝒜)=0. (11)

This work is motivated by the ongoing analysis of various generalizations of operator classes, as cited above, particularly those related to Helton-type operators. In this context, we aim to introduce the polynomially quasi-Helton class of order *m*, which extends and unifies two important existing notions: the Helton class of order *m*. and the *n*-quasi-Helton class of order *m*. The new class is designed to provide a broader framework for studying higher-order commutator relationships and structural properties within the theory of bounded linear operators on Hilbert spaces. We invite the reader to refer to the references [8,19–23] for more details about other interesting classes of operators in this context.

## 2. Main results

The main properties of this new class of operators are introduced and studied in this section.

**Definition 2.1.** *For a positive integer m and* 𝒜,ℬ∈ℒ(𝒦)*, we say that* 𝒜 *belongs to polynomially-quasi Helton class of* ℬ *with order m if there exists a nonconstant polynomial* 𝒫 *such that*


$$\begin{array}{c} 𝒫(𝒜{)}^{*}\Phi (ℬ;𝒜{)}^{m}(ℐ)𝒫(𝒜)=0, \end{array}$$ (12)

*which can be expressed analytically as*


𝒫(𝒜)*(∑0≤j≤m(−1)j(mj)ℬj𝒜m−j)𝒫(𝒜)=0. (13)

*This is symbolized by* $𝒜\in [𝒬{HEL}_{\left(m,𝒫\right)}(ℬ)]$.

**Remark 2.2.**

(i) If 𝒫(x)=x, the class $\left[𝒬{HEL}_{\left(m,𝒫\right)}(B)\right]$ reduces to the classical quasi-Helton class of order *m*.
(ii) If $𝒫(x)=x^{n}$, the class $\left[𝒬{HEL}_{\left(m,𝒫\right)}(ℬ)]=[n𝒬]\cap [{HEL}_{m}(ℬ)\right]$.
(iii) If $𝒜\in [{HEL}_{m}(ℬ)]$ then $𝒜\in [{QHEL}_{\left(m,𝒫\right)}(ℬ)].$

**Example 2.3.** *We construct an example of two operators* A*,* $ℬ\in {\mathbb{C}}^{3\times 3}$*, and a polynomial* 𝒫∈ℂ[z]*, such that:*


$$\begin{array}{c} 𝒫(𝒜{)}^{*}(ℬ-𝒜)𝒫(𝒜)=0\;\text{but}\;{𝒜}^{*}(ℬ-𝒜)𝒜\neq 0 \end{array}$$

*with the additional condition that* $P(A)\neq A^{k}$ *for any* k∈ℕ.

*Let*


$$\begin{array}{c} 𝒜=[\begin{array}{ccc} 0 & 1 & 0\\ 0 & 0 & 1\\ 0 & 0 & 0 \end{array}],\;M=[\begin{array}{ccc} 1 & 0 & 0\\ 0 & 0 & 0\\ 0 & 0 & 0 \end{array}],\;ℬ=𝒜+M. \end{array}$$

*Let*

$𝒫(z)=z^{2}+z\;\Rightarrow \;𝒫(𝒜)=A^{2}+𝒜.$ *We compute:*


$$\begin{array}{c} {𝒜}^{2}=[\begin{array}{ccc} 0 & 0 & 1\\ 0 & 0 & 0\\ 0 & 0 & 0 \end{array}],\;𝒜=[\begin{array}{ccc} 0 & 1 & 0\\ 0 & 0 & 1\\ 0 & 0 & 0 \end{array}]\;\Rightarrow \;𝒫(𝒜)={𝒜}^{2}+𝒜=[\begin{array}{ccc} 0 & 1 & 1\\ 0 & 0 & 1\\ 0 & 0 & 0 \end{array}]. \end{array}$$

$$\begin{array}{c} M𝒫(𝒜)=0\;\Rightarrow \;𝒫(𝒜{)}^{*}(ℬ-𝒜)𝒫(𝒜)=𝒫(𝒜{)}^{*}M𝒫(𝒜)=0. \end{array}$$

*However,* ${𝒜}^{*}(ℬ-𝒜)A={𝒜}^{*}M𝒜\neq 0$*. Therefore:*


$$\begin{array}{c} {𝒜}^{*}(ℬ-𝒜)𝒜\neq 0. \end{array}$$

*We have constructed an example with:*

- *A polynomial* $𝒫(z)=z^{2}+z\notin \{z^{k}\}.$
- *Operators* 𝒜*,* ℬ *such that:*
  $$\begin{array}{c} P(𝒜{)}^{*}(ℬ-𝒜)𝒫(𝒜)=0\;\text{and}\;{𝒜}^{*}(ℬ-𝒜)𝒜\neq 0. \end{array}$$

**Example 2.4.** *(Composition operator with polynomial* $P(z)=z^{2}-1$*). Let* $𝒦=H^{2}(𝔻)$*, the Hardy space on the unit disk. Define the composition operator*


$$\begin{array}{c} {𝒞}_{\phi }f=f\circ \phi ,\;f\in H^{2}(𝔻), \end{array}$$

*with symbol* $\phi (z)=\frac{z}{2}$.

*Define*


$$\begin{array}{c} ℬ={𝒞}_{\phi }+I,\;P(z)=z^{2}-1. \end{array}$$

*Step 1: Helton expression.*

*For* m≥1*, the Helton expression is*


Φ(ℬ;𝒞ϕ)m=∑j=0m(−1)j(mj)ℬj𝒞ϕm−j.

*Since* $ℬ={𝒞}_{\phi }+I$ *and composition operators commute with their powers, we can expand:*


ℬj𝒞ϕm−j=∑k=0j(jk)𝒞ϕj−k𝒞ϕm−j=∑k=0j(jk)𝒞ϕm−k.

*Hence*


Φ(ℬ;𝒞ϕ)m=∑k=0m(∑j=km(−1)j(mj)(jk))𝒞ϕm−k.

*Using the standard combinatorial identity, only the term k = m survives:*


$$\begin{array}{c} \Phi (ℬ;{𝒞}_{\phi }{)}^{m}=(-1{)}^{m}I. \end{array}$$

*Step 2: PQ-Helton condition.*

*Compute*


$$\begin{array}{c} 𝒫({𝒞}_{\phi }{)}^{*}\Phi (ℬ;{𝒞}_{\phi }{)}^{m}𝒫({𝒞}_{\phi })=(-1{)}^{m}𝒫(C_{\phi }{)}^{*}𝒫(C_{\phi }). \end{array}$$

*Since* $𝒫({𝒞}_{\phi })={𝒞}_{\phi }^{2}-I$ *is non-zero but has a nontrivial kernel (functions satisfying* $f\circ \phi^{2}=f$*), the operator*


$$\begin{array}{c} 𝒫({𝒞}_{\phi }{)}^{*}\Phi (ℬ;{𝒞}_{\phi }{)}^{m}P({𝒞}_{\phi }) \end{array}$$

*vanishes on a nontrivial subspace. This satisfies the polynomially-quasi Helton condition non-trivially. Thus, the composition operator* ${𝒞}_{\phi }\in [{QHEL}_{\left(m,𝒫\right)}(ℬ)]$*, providing a non-trivial infinite-dimensional example in the operator theoretic setting.*

**Definition 2.5.** *For a positive integer* m≥2 *and* 𝒜,ℬ∈ℒ(𝒦)*, we say that* 𝒜 *belongs to polynomially-quasi strict Helton class of* ℬ *with order* m≥ *if there exists a nonconstant polynomial* 𝒫 *such that* $𝒜\in [𝒬{HEL}_{\left(m,𝒫\right)}(ℬ)]$ *and* $𝒜\notin [𝒬{HEL}_{\left(m-1,𝒫\right)}(ℬ)]$*, that is*


$$\begin{array}{c} 𝒫(𝒜{)}^{*}\Phi (ℬ;𝒜{)}^{m}(ℐ)𝒫(𝒜)=0\;\;\text{and}\;\;𝒫(𝒜{)}^{*}\Phi (ℬ;𝒜{)}^{m-1}(ℐ)𝒫(𝒜)\neq 0. \end{array}$$

**Example 2.6.** *We are looking for operators* 𝒜*,* ℬ*, and a polynomial* $𝒫(z)\neq z^{k}$ *such that:*


$$\begin{array}{cc} \text{(1)}\; & 𝒫(𝒜{)}^{*}(ℬ-𝒜)𝒫(𝒜)\neq 0\\ \text{(2)}\; & 𝒫(𝒜{)}^{*}({ℬ}^{2}-2ℬ𝒜+{𝒜}^{2})P(𝒜)=0. \end{array}$$

*In fact, let us define the following matrices:*


$$\begin{array}{c} 𝒜=[\begin{array}{ccc} 0 & 1 & 0\\ 0 & 0 & 0\\ 0 & 0 & 0 \end{array}],\;B=[\begin{array}{ccc} 0 & 1 & 1\\ 0 & 0 & 0\\ 0 & 0 & 0 \end{array}]\;\;\mathrm{and let the polynomial be}:𝒫(z)=z+1. \end{array}$$

*We verify the conditions:*

- *Compute* 𝒫(𝒜)=𝒜+ℐ*, and* $𝒫(𝒜{)}^{*}=(𝒜+ℐ{)}^{*}$:
  $$\begin{array}{c} P(𝒜)=[\begin{array}{ccc} 1 & 1 & 0\\ 0 & 1 & 0\\ 0 & 0 & 1 \end{array}],\;P(𝒜{)}^{*}=[\begin{array}{ccc} 1 & 0 & 0\\ 1 & 1 & 0\\ 0 & 0 & 1 \end{array}]. \end{array}$$

*Compute* ℬ−A:


$$\begin{array}{c} B-𝒜=[\begin{array}{ccc} 0 & 0 & 1\\ 0 & 0 & 0\\ 0 & 0 & 0 \end{array}]. \end{array}$$

*Then:*


$$\begin{array}{c} 𝒫(𝒜{)}^{*}(ℬ-𝒜)𝒫(𝒜)=[\begin{array}{ccc} 1 & 0 & 0\\ 1 & 1 & 0\\ 0 & 0 & 1 \end{array}][\begin{array}{ccc} 0 & 0 & 1\\ 0 & 0 & 0\\ 0 & 0 & 0 \end{array}][\begin{array}{ccc} 1 & 1 & 0\\ 0 & 1 & 0\\ 0 & 0 & 1 \end{array}]=[\begin{array}{ccc} 0 & 0 & 1\\ 0 & 0 & 1\\ 0 & 0 & 0 \end{array}]\neq 0 \end{array}$$

*and*

- $𝒫(𝒜{)}^{*}({ℬ}^{2}-2ℬ𝒜+{𝒜}^{2})𝒫(𝒜)=𝒫(𝒜{)}^{*}(ℬ(ℬ-𝒜)-(ℬ-𝒜)𝒜)𝒫(𝒜)=0$.

*So, conditions (1) and (2) are satisfy.*

**Proposition 2.7.** *Let* 𝒫∈ℂ[z]⧵{0} *and let* 𝒜,ℬ∈ℒ(𝒦)*. Suppose that* $𝒜\in [𝒬{HEL}_{\left(m,𝒫\right)}(ℬ)]$*. If* R(𝒫(𝒜)) *is dense. Then* $𝒜\in [{HEL}_{m}(ℬ)].$

*Proof.* Set


M:=Φ(ℬ;𝒜)m(ℐ)=∑j=0m(−1)j(mj)ℬj𝒜m−j.

Since $𝒜\in [𝒬{HEL}_{\left(m,𝒫\right)}(ℬ)]$ there exists a nonzero polynomial 𝒫∈C[x] for which


$$\begin{array}{c} 𝒫(𝒜{)}^{*}\;M\;𝒫(𝒜)=0. \end{array}$$ (14)

From (14) we obtain the range-kernel inclusion


$$\begin{array}{c} \mathrm{ran}(M𝒫(𝒜))\subseteq \ker\;(𝒫(𝒜{)}^{*}), \end{array}$$

By hypothesis ran𝒫(𝒜) is dense in 𝒦. Hence


$$\begin{array}{c} \ker\;(𝒫(𝒜{)}^{*})=(\overset{―}{\mathrm{ran}𝒫(𝒜)}{)}^{⟂}=\{0\}. \end{array}$$

Therefore ran(M𝒫(𝒜))={0}, i.e.,


$$\begin{array}{c} M𝒫(𝒜)=0. \end{array}$$

Because ran𝒫(𝒜) is dense and *M* is bounded, the identity M𝒫(𝒜)=0 implies *M* = 0 on the whole space (a bounded operator which vanishes on a dense subspace is identically zero). Hence


$$\begin{array}{c} M=0. \end{array}$$

Thus 𝒜 satisfies the Helton-type identity of order *m*, i.e., $𝒜\in [{HEL}_{m}(ℬ)]$, as claimed.

**Proposition 2.8.** *Let* 𝒫∈ℂ[z]⧵{0} *and let* $𝒜\in [𝒬{HEL}_{\left(m,𝒫\right)}(ℬ)]$*. If* $\ker({𝒜}^{*})=\ker(𝒫(𝒜){)}^{*})$*, then* $𝒜\in [𝒬]\cap [{HEL}_{m}(ℬ)]$*.*

*Proof.* Let


M:=Φ(ℬ;𝒜)m(ℐ)=∑j=0m(−1)j(mj)ℬj𝒜m−j.

Assume that $𝒜\in [𝒬{HEL}_{\left(m,𝒫\right)}(ℬ)]$. By definition, there exists a nonzero polynomial 𝒫 such that


$$\begin{array}{c} 𝒫(𝒜{)}^{*}\;M\;𝒫(𝒜)=0. \end{array}$$ (15)

We use the elementary fact that for bounded operators *S*,*T*, if $S^{*}T=0$, then $\mathrm{ran}T\subseteq \ker S^{*}$. Applying this to (15), we obtain


$$\begin{array}{c} \mathrm{ran}(M𝒫(𝒜))\subseteq \ker\;(𝒫(𝒜{)}^{*}). \end{array}$$

Since by hypothesis


$$\begin{array}{c} \ker({𝒜}^{*})=\ker\;(𝒫(𝒜{)}^{*}), \end{array}$$

it follows that


$$\begin{array}{c} \mathrm{ran}(M𝒫(𝒜))\subseteq \ker({𝒜}^{*}), \end{array}$$

which is equivalent to


$$\begin{array}{c} {𝒜}^{*}M\;𝒫(𝒜)=0. \end{array}$$ (16)

Taking adjoints in (16) yields


$$\begin{array}{c} 𝒫(𝒜{)}^{*}M^{*}𝒜=0. \end{array}$$ (17)

Applying the same range-kernel argument to (17), we obtain


$$\begin{array}{c} \mathrm{ran}(M^{*}𝒜)\subseteq \ker\;(𝒫(𝒜{)}^{*})=\ker({𝒜}^{*}), \end{array}$$

so that


$$\begin{array}{c} {𝒜}^{*}M^{*}𝒜=0. \end{array}$$ (18)

Taking adjoints of (18) (or repeating the same argument with *M* in place of $M^{*}$) gives


$$\begin{array}{c} {𝒜}^{*}M𝒜=0. \end{array}$$ (19)

Therefore,


$$\begin{array}{c} 𝒜\in [𝒬]\cap [{HEL}_{m}(ℬ)], \end{array}$$

as required.

It was proved in [24] that if $𝒜\in [{HEL}_{m}(ℬ)]$ for some positive integer m≥1, then $𝒜{|}_{ℳ}\in {HEL}_{m}(ℬ{|}_{ℳ})$ whenever ℳ∈Lat(𝒜)∩Lat(ℬ), where Lat(𝒜) denotes the lattice of all invariant subspaces for 𝒜. In the following theorem, we generalize this result to a broader subclass within the framework considered in $\left[𝒬{HEL}_{\left(m,𝒫\right)}(ℬ)\right]$.

**Theorem 2.9.** *Let* 𝒜,ℬ∈ℒ(𝒦) *and let* 𝒫∈C[z]⧵{0}*. Assume* $𝒜\in [𝒬{HEL}_{\left(m,𝒫\right)}(ℬ)].$ *Let* ℳ *be a closed subspace of* 𝒦 *which reduces* ℬ *(i.e.,* ℳ *is invariant for both* B *and* $B^{*}$) and is invariant for A*. Then with respect to the orthogonal decomposition* $𝒦=ℳ⊕{ℳ}^{⟂}$ *we have the block upper-triangular representations*


$$\begin{array}{c} 𝒜=(\begin{array}{cc} {𝒜}_{1} & {𝒜}_{2}\\ 0 & {𝒜}_{3} \end{array}),\;ℬ=(\begin{array}{cc} {ℬ}_{1} & {ℬ}_{2}\\ 0 & {ℬ}_{3} \end{array}), \end{array}$$

*and therefore*


$$\begin{array}{c} {ℬ}^{*}=(\begin{array}{cc} {ℬ}_{1}^{*} & 0\\ {ℬ}_{2}^{*} & {ℬ}_{3}^{*} \end{array}). \end{array}$$

*Moreover, the compression of the polynomially quasi-m-Helton identity to* M *shows*


$$\begin{array}{c} {𝒜}_{1}\in [𝒬{HEL}_{\left(m,𝒫\right)}({ℬ}_{1})] \end{array}$$

*where* ${𝒜}_{1}=𝒜{|}_{ℳ}$ *and* ${ℬ}_{1}=ℬ{|}_{ℳ}$.

*Proof.* Set


M:=Φ(B;A)m(I)=∑j=0m(−1)j(mj)BjAm−j.

By hypothesis there is a nonzero polynomial P with


$$\begin{array}{c} P(A{)}^{*}\;M\;P(A)=0. \end{array}$$ (20)

Block decompositions. Since M is invariant for A and reduces B, write $K=M⊕M^{⟂}$ and use the induced block form for A and B:


$$\begin{array}{c} A=(\begin{array}{cc} A_{1} & A_{2}\\ 0 & A_{3} \end{array}),\;B=(\begin{array}{cc} B_{1} & B_{2}\\ 0 & B_{3} \end{array}). \end{array}$$

Because M reduces B, powers of B also have the same block upper-triangular structure:


$$\begin{array}{c} B^{j}=(\begin{array}{cc} B_{1}^{j} & *\\ 0 & B_{3}^{j} \end{array}),\;j\geq 0, \end{array}$$

and since M is invariant for A, powers of A satisfy


$$\begin{array}{c} A^{\;k}=(\begin{array}{cc} A_{1}^{\;k} & *\\ 0 & A_{3}^{\;k} \end{array}),\;k\geq 0. \end{array}$$

Polynomials of A. Since M is invariant under A, every polynomial in A leaves M invariant. Hence the restricted operator $P(A){|}_{M}$ equals $P(A_{1})$ (the polynomial evaluated at the block $A_{1}$). In block form,


$$\begin{array}{c} P(A)=(\begin{array}{cc} P(A_{1}) & *\\ 0 & P(A_{3}) \end{array}). \end{array}$$

Compression of the Helton relation to M. Write the block form of *M*. Using the block forms of $B^{j}$ and $A^{m-j}$ and collecting diagonal (M→M) terms, the diagonal (M→M) block of *M* is exactly


M11=∑j=0m(−1)j(mj)B1jA1m−j=Φ(B1;A1)m(IM).

Now take the (1,1) (i.e., M→M) block of the operator identity (20). Because


$$\begin{array}{c} P(A{)}^{*}=(\begin{array}{cc} P(A_{1}{)}^{*} & 0\\ {}* & P(A_{3}{)}^{*} \end{array}),\;M=(\begin{array}{cc} M_{11} & *\\ 0 & * \end{array}), \end{array}$$

the (1,1) block of $P(A{)}^{*}MP(A)$ equals


$$\begin{array}{c} P(A_{1}{)}^{*}\;M_{11}\;P(A_{1}). \end{array}$$

Since the whole operator in (20) is zero, its (1,1) block vanishes, hence


$$\begin{array}{c} P(A_{1}{)}^{*}\;\Phi (B_{1};A_{1}{)}^{m}(I_{M})\;P(A_{1})=0. \end{array}$$

Thus $A_{1}\in [𝒬{HEL}_{\left(m,P\right)}(B_{1})]$. This completes the proof.

**Theorem 2.10.** *Let* 𝒜,ℬ∈L[K] *and* 𝒫∈ℂ[z]⧵{0}*. If* 𝒫(𝒜)≠0*,* $\overset{―}{ran(𝒫(𝒜))}\neq 𝒦$ *and* $\overset{―}{ran(𝒫(𝒜))}$ *reduces* ℬ*. The equivalence of the following statements holds true:*

(1) $𝒜\in [𝒬{HEL}_{\left(m,𝒫\right)}(ℬ)]$.
(2) 𝒜 *and* ℬ *have upper triangular representations*
  $$\begin{array}{c} 𝒜=(\begin{array}{cc} {𝒜}_{1} & {𝒜}_{2}\\ 0 & {𝒜}_{3} \end{array}),\;ℬ=(\begin{array}{cc} {ℬ}_{1} & 0\\ 0 & {ℬ}_{2} \end{array})\;\text{on }𝒦=\overset{―}{ran(𝒫(𝒜))}⊕\ker(𝒫(𝒜{)}^{*}), \end{array}$$
  *where* ${𝒜}_{1}\in [{HEL}_{m}({ℬ}_{1})],𝒫({𝒜}_{3})=0$ *and where* ${𝒜}_{1}=𝒜{|}_{ℳ}$ *and* ${ℬ}_{1}=ℬ{|}_{ℳ}$ *with* $ℳ=\overset{―}{ran(𝒫(𝒜))}$.

*Proof.* Since $𝒜(\overset{―}{ran(𝒫(𝒜)})\subseteq \overset{―}{ran(𝒫(𝒜)})$, ${ℬ}^{*}(\overset{―}{ran(𝒫(𝒜))}\subseteq \overset{―}{ran(𝒫(𝒜)})$ and $ℬ(\overset{―}{ran(𝒫(𝒜)})\subseteq \overset{―}{ran(𝒫(𝒯)})$, we may write $ℬ={ℬ}_{1}⊕{ℬ}_{2}$ with ${ℬ}_{1}=ℬ{|}_{\overset{―}{ran(𝒫(𝒜))}}$ and ${ℬ}_{2}=ℬ{|}_{\ker(𝒫(𝒜{)}^{*})}$ and 𝒜 has a matrix decomposition $𝒜=\left(\begin{array}{cc} {𝒜}_{1} & {𝒜}_{2}\\ 0 & {𝒜}_{3} \end{array}\right)$, relative to $K=\overset{―}{ran(𝒫)(𝒜)}⊕\ker(𝒫(𝒜{)}^{*})$.

Similarly, ℬ has a matrix decomposition $ℬ=\left(\begin{array}{cc} {ℬ}_{1} & 0\\ 0 & {ℬ}_{2} \end{array}\right)$, relative to $K=\overset{―}{ran(𝒫)(𝒜)}⊕N(𝒫(𝒜{)}^{*})$.

Let ℙ denote the projection of K onto $\overset{―}{ran(𝒫(𝒜))}$. As $𝒜\in [{QHEL}_{\left(m,𝒫\right)}(ℬ)]$, we have


𝒫(𝒜)*(∑0≤k≤m(−1)k(mk)ℬk𝒜m−k)𝒫(𝒜)=0,

This can be expressed as


⟨𝒫(𝒜)*(∑0≤k≤m(−1)k(mk)ℬ*k𝒜m−k)𝒫(𝒜)ω∣ω⟩=0∀ω∈𝒦.

We have the ability to write


⟨𝒫(𝒜)*(∑0≤k≤m(−1)k(mk)ℬk𝒜m−k)𝒫(𝒜)ω∣ω⟩=0∀ω∈𝒦,⟹⟨(∑0≤k≤m(−1)k(mk)ℬk𝒜m−k)𝒫(𝒜)ω∣𝒫(𝒜)ω⟩=0∀ω∈𝒦,⟹⟨(∑0≤k≤m(−1)k(mk)ℬk𝒜m−k)ψ∣ψ⟩=0∀ψ∈ran(𝒫(𝒜))―⟹⟨(∑0≤k≤m(−1)k(mk)ℬk𝒜m−k)ℙω∣ℙω⟩=0∀ω∈K(sinceℙω∈ran(𝒫(𝒜))―)⟹⟨ℙ(∑0≤k≤m(−1)k(mk)ℬk𝒜m−k)ℙω∣ω⟩=0∀ω∈K⟹ℙ(∑0≤k≤m(−1)k(mk)ℬk𝒜m−k)ℙ=0.

Taking into consideration that $\mathbb{P}=\left(\begin{array}{cc} {ℐ}_{1} & 0\\ 0 & 0 \end{array}\right),I_{1}=ℐ{|}_{\overset{―}{ran(𝒫(𝒜))}},$


$$\begin{array}{c} ℬ=\left(\begin{array}{cc} {ℬ}_{1} & 0\\ 0 & {ℬ}_{2} \end{array}\right)\;\text{and}\;\;𝒜=\left(\begin{array}{cc} {𝒜}_{1} & {𝒜}_{2}\\ 0 & A_{3} \end{array}\right). \end{array}$$

By conducting simple calculations, we find that


ℙ(∑0≤k≤m(−1)k(mk)ℬk𝒜m−k)ℙ=(∑0≤k≤m(−1)k(mk)ℬ1k𝒜1m−k000)

Therefore, ∑0≤k≤m(−1)k(mk)ℬ1k𝒜1n−k=0. Hence ${𝒜}_{1}\in [{HEL}_{m}({ℬ}_{1})].$

Let $\psi =\psi_{1}+\psi_{2}\in K=\overset{―}{ran(𝒫(𝒜))}⊕\ker(𝒫(𝒜{)}^{*})$.

By explicit calculation, we obtain that


$$\begin{array}{cc} \langle 𝒫({𝒜}_{3})\psi_{2}|\psi_{2}\rangle & =\langle 𝒫(𝒜)(ℐ-\mathbb{P})\psi |(ℐ-\mathbb{P})\psi \rangle \\ & =\langle (ℐ-𝒫)\psi |𝒫(𝒜{)}^{*}(ℐ-\mathbb{P})\psi \rangle =0. \end{array}$$

So, $𝒫({𝒜}_{3})=0.$

(2) implies (1). Let $𝒜=\left(\begin{array}{cc} {𝒜}_{1} & {𝒜}_{2}\\ 0 & {𝒜}_{3} \end{array}\right)$ and $ℬ=\left(\begin{array}{cc} {ℬ}_{1} & 0\\ 0 & {ℬ}_{2} \end{array}\right)$

onto $K=\overset{―}{ℛ(𝒫)(𝒜)}⊕\ker(𝒫(𝒜{)}^{*})$, with


Φ(ℬ1,𝒜1)m:=∑0≤k≤m(−1)k(mk)ℬ1k𝒜1m−k=0and𝒫(𝒜3)=0.

Since ${𝒜}^{k}=\left(\begin{array}{cc} {𝒜}_{1}^{k} & \sum_{j=0}^{k-1}{𝒜}_{1}^{j}{𝒜}_{2}{𝒜}_{3}^{k-1-j}\\ 0 & {𝒜}_{3}^{k} \end{array}\right)$ and ${ℬ}^{k}=\left(\begin{array}{cc} {ℬ}_{1}^{k} & 0\\ 0 & {ℬ}_{2}^{k} \end{array}\right)$ it can be seen that


𝒫(𝒜)*(∑0≤k≤m(−1)k(mk)ℬk𝒜m−k)𝒫(𝒜)=(𝒫(𝒜1)Δ0𝒫(𝒜3))*{I+∑1≤k≤m(ℬ100ℬ2)k)(𝒜1𝒜20𝒜3)m−k}×(𝒫(𝒜1)Δ0𝒫(𝒜3))

=(𝒫(𝒜1)*0Δ*𝒫(𝒜3)*)×{I+∑1≤k≤m(−1)k(mk)(ℬ1k00ℬ2k)(𝒜1k∑j=0m−k−1𝒜1j𝒜2𝒜3m−k−1−j0𝒜3m−k)}×(P(𝒜1)Δ0𝒫(𝒜3))=(𝒫(𝒜1)*0Δ*0)×{(Φ(ℬ1,𝒜1)m𝒞DE)}×(𝒫(𝒯1)Δ00)

$$\begin{array}{cc} & =\left(\begin{array}{cc} 𝒫({𝒜}_{1}{)}^{*}\Phi ({ℬ}_{1},{𝒜}_{1}{)}^{m}𝒫({𝒜}_{1}) & 𝒫({𝒜}_{1}{)}^{*}\Phi ({ℬ}_{1},{𝒜}_{1}{)}^{m}\Omega \\ \Delta^{*}\Phi ({ℬ}_{1},{𝒜}_{1}{)}^{m}𝒫({𝒜}_{1}) & \Delta^{*}\Phi ({ℬ}_{1},{𝒜}_{1}{)}^{m}\Delta \end{array}\right)\\ & =\left(\begin{array}{cc} 0 & 0\\ 0 & 0 \end{array}\right). \end{array}$$

Hence $𝒫(𝒜)\Phi (ℬ,𝒜{)}^{m}(ℐ)𝒫(𝒜)=0$. This concludes the proof.

It was proved in [11, Lemma 2.8] that if $𝒜\in [{HEL}_{m}(ℬ)]$ for some positive integer m≥2, then ${𝒜}^{n}\in [{HEL}_{m}({ℬ}^{n})]$ for positive integer *n*. In the following corollary, we generalize this result to a broader subclass within the framework considered in $\left[𝒬{HEL}_{\left(m,𝒫\right)}(ℬ)\right]$.

**Corollary 2.11.** *Let* 𝒫∈ℂ[x]⧵{0} *and let* 𝒜,ℬ∈ℒ(𝒦) *satisfy the same hypotheses as in Theorem 2.10. If* $𝒜\in [{QHEL}_{\left(m,𝒫\right)}(ℬ)]$*, then* ${𝒜}^{n}\in [{QHEL}_{\left(m,𝒫\right)}({ℬ}^{n})]$*for positive integer n and any integer* m≥2.

*Proof.* We divide the proof into two steps, based on the assumption that $𝒜\in [{QHEL}_{\left(m,𝒫\right)}(ℬ)]$.:

Step 1: If $\overset{―}{ran(𝒫(𝒜))}=𝒦$, then $𝒜\in [{HEL}_{m}(ℬ)]$ by Proposition 2.7. Therefore, ${𝒜}^{n}\in [{HEL}_{m}({ℬ}^{n})]$ for positive integer *n* in view of [11, Lemma 2.8].

Step 2: If $\overset{―}{ran(𝒫(𝒜))}\text{≠}𝒦.$ By Theorem 2.10, we obtain $𝒜=\left(\begin{array}{cc} {𝒜}_{1} & {𝒜}_{2}\\ 0 & {𝒜}_{3} \end{array}\right)$ and $ℬ=\left(\begin{array}{cc} {ℬ}_{1} & 0\\ 0 & {ℬ}_{2} \end{array}\right)$ on $𝒦=\overset{―}{ran(𝒫(𝒜))}⊕\ker((𝒫(𝒜){)}^{*})$, where ${𝒜}_{1}\in [{HEL}_{m}({ℬ}_{1})]$ and $𝒫({𝒜}_{3})=0.$

We proceed by analyzing the case where ${𝒜}^{n}=\left(\begin{array}{cc} {𝒜}_{1}^{n} & ⋆\\ 0 & {𝒜}_{3}^{n} \end{array}\right)$ and ${ℬ}^{n}=\left(\begin{array}{cc} {ℬ}_{1}^{n} & 0\\ 0 & {ℬ}_{2}^{n} \end{array}\right).$ Given that ${𝒜}_{1}\in [{HEL}_{m}({ℬ}_{1})]$, it follows that ${𝒜}_{1}^{n}\in [{HEL}_{m}({ℬ}_{1}^{n})]$ by the statement of [11, Lamma 2.8]. Despite that ${𝒜}_{3}^{n}$ is algebraic. By virtue of Theorem 2.10, we conclude that ${𝒜}^{n}\in [{QHEL}_{\left(m,𝒫\right)}({ℬ}^{n})]$.This establishes the result. The single-valued extension property (SVEP) is a standard concept in local spectral theory. It has been studied and developed by many authors.

**Definition 2.12. (Single-Valued Extension Property (SVEP)** [25]**).** *Let* 𝒜∈ℒ(𝒦) *and let* λ∈ℂ*.* 𝒜 *is said to have has the Single-Valued Extension Property (SVEP) at the point* λ *if: For every neighborhood U of* λ*, and for every analytic function* f:U→𝒦 *such that* (𝒜−μℐ)f(μ)=0for all μ∈U, *we necessarily have* f(μ)=0 *for all* μ∈U*. If* 𝒰 *has SVEP at every* λ∈ℂ*, we say simply that* 𝒜 *has SVEP.*

Let B∈ℒ(𝒦) have the single-valued extension property. If $𝒜\in [{HEL}_{m}(ℬ)]$, then 𝒜 also has the single-valued extension property. This result was established [16, Theorem 2]. The next proposition gives a result of the same nature for operators belonging to class $[{QHEL}_{(m,𝒫}(ℬ)].$

**Theorem 2.13.** *(*[26]*) Suppose that* $T_{i}\in ℒ(X_{i})$ *for i = 1, 2, where* $X_{i}$ *are Banach spaces. Then* $T_{1}⊕T_{2}$ *has the single-valued extension property (SVEP) at* $\lambda_{0}$ *if and only if both T*_*1*_ *and T*_*2*_ *have the SVEP at* $\lambda_{0}$*.*

**Proposition 2.14.** *Let* 𝒫∈ℂ[z]⧵{0} *and let* 𝒜,ℬ∈ℒ(𝒦) *satisfy the same hypotheses as in Theorem 2.10. If* ℬ *has SEVP and* $𝒜\in [{QHEL}_{\left(m,𝒫\right)}(ℬ)]$*, then* 𝒜 *has SVEP.*

*Proof.* Let $𝒜\in [{QHEL}_{\left(m,𝒫\right)}(ℬ)]$ and assume that ℬ has SVEP.

**Case (1)**: $\overset{―}{ℛ(𝒫(𝒜))}=𝒦$

In this case, since 𝒫(𝒜) has dense range, it follows from Proposition 2.7, that


$$\begin{array}{c} 𝒜\in [{HEL}_{m}(ℬ)]. \end{array}$$

By [[27], Theorem 2], any Helton operator of order *m* with respect to an operator having SVEP also has SVEP. Hence, 𝒜 has SVEP.

**Case (2)**: $\overset{―}{ℛ(𝒫(𝒜))}\neq 𝒦$

By Theorem 2.10, there exists a block decomposition with respect to


$$\begin{array}{c} 𝒦=\overset{―}{ran(𝒫(𝒜))}⊕\ker(𝒫(𝒜{)}^{*}), \end{array}$$

such that


$$\begin{array}{c} 𝒜=(\begin{array}{cc} {𝒜}_{1} & {𝒜}_{2}\\ 0 & {𝒜}_{3} \end{array}),\;ℬ=(\begin{array}{cc} {ℬ}_{1} & 0\\ 0 & {ℬ}_{2} \end{array}),\;𝒫({𝒜}_{3})=0. \end{array}$$

Let $f(\lambda )=f_{1}(\lambda )+f_{2}(\lambda )$ be an analytic function with $f_{1}(\lambda )\in \overset{―}{ran(𝒫(𝒜))}$ and $f_{2}(\lambda )\in \ker(𝒫(𝒜{)}^{*})$, satisfying


$$\begin{array}{c} (𝒜-\lambda I)f(\lambda )=0. \end{array}$$

Then we have


$$\begin{array}{c} 0=(𝒜-\lambda I)(\begin{array}{c} f_{1}(\lambda )\\ f_{2}(\lambda ) \end{array})=(\begin{array}{c} \left({𝒜}_{1}-\lambda I)f_{1}(\lambda )+{𝒜}_{2}f_{2}(\lambda \right)\\ \left({𝒜}_{3}-\lambda I)f_{2}(\lambda \right) \end{array}). \end{array}$$

This gives the system:


$$\begin{array}{c} \left\{ \begin{array}{c} ({𝒜}_{1}-\lambda I)f_{1}(\lambda )+{𝒜}_{2}f_{2}(\lambda )=0,\\ ({𝒜}_{3}-\lambda I)f_{2}(\lambda )=0. \end{array}\right. \end{array}$$

In view of theorem 2.13, ${ℬ}_{2}$ has SVEP and ${𝒜}_{3}\in [{QHEL}_{\left(m,𝒫\right)}({ℬ}_{2})]$ on $\ker(𝒫(𝒜{)}^{*})$, it follows that ${𝒜}_{3}$ has SVEP (or by algebraic operator argument (see [25]). Hence, the second equation implies


$$\begin{array}{c} f_{2}(\lambda )=0. \end{array}$$

Substituting back into the first equation, we obtain


$$\begin{array}{c} ({𝒜}_{1}-\lambda I)f_{1}(\lambda )=0. \end{array}$$

By the argument used in Case (1), ${𝒜}_{1}$ inherits SVEP from ${ℬ}_{1}$, which has SVEP (Theorem 2.13). Therefore, $f_{1}(\lambda )=0$.

Combining both, we have


$$\begin{array}{c} f(\lambda )=f_{1}(\lambda )+f_{2}(\lambda )=0. \end{array}$$

Consequently, 𝒜 has the Single Valued Extension Property (SVEP).

**Proposition 2.15.** *Let* 𝒫∈ℂ[z]⧵{0} *and let* 𝒜,ℬ∈ℒ(𝒦)*. The following properties hold.*

(1) $𝒜\in [{QHEL}_{\left(m,𝒫\right)}(ℬ)]$ *if and only if* ${ℰ}^{-1}𝒜ℰ\in [{QHEL}_{\left(m,𝒫\right)}({ℰ}^{-1}ℬℰ)]$ *for all invertible* ℰ∈ℒ(𝒦).
(2) *If* ${𝒜}_{k}\in [𝒬{HEL}_{\left(m,𝒫\right)}({ℬ}_{k})]$ *for k = 1,2, then* ${𝒜}_{1}⊕{𝒜}_{2}\in [𝒬{HEL}_{\left(m,𝒫\right)}({ℬ}_{1}⊕{ℬ}_{2})].$

*Proof.* (i) Clearly, we have


𝒫(ℰ−1𝒜ℰ)*Φ(ℰ−1ℬℰ;ℰ−1𝒜ℰ)m(ℐ)𝒫(ℰ−1𝒜ℰ)=ℰ−1𝒫(𝒜)*ℰ(∑0≤j≤m(−1)j(mj)(ℰ−1ℬℰ)j(ℰ−1Aℰ)m−j)𝒫(ℰ−1𝒜ℰ)=ℰ−1𝒫(𝒜)(∑0≤j≤m(−1)j(mj)ℬj𝒜m−j)𝒫(𝒜)ℰ=ℰ−1𝒫(𝒜)Φ(ℬ;𝒜)𝒫(𝒜)ℰ.

Therefore,


$$\begin{array}{c} 𝒜\in [{QHEL}_{\left(m,𝒫\right)}(ℬ)]⟺{ℰ}^{-1}𝒜ℰ\in [{QHEL}_{\left(m,𝒫\right)}({ℰ}^{-1}ℬℰ)]. \end{array}$$

(2) We have $𝒫({𝒜}_{1}⊕𝒫({𝒜}_{2})=𝒫({𝒜}_{1})⊕{𝒜}_{2}).$ Now from that ${𝒜}_{i}\in [𝒬{HEL}_{\left(m,𝒫\right)}({ℬ}_{i})]$ for *i* = 1,2 and 𝒫(0)≠0, we get


𝒫(𝒜1⊕𝒜2)*Φ(ℬ1⊕ℬ2;𝒜1⊕𝒜2)m(I)𝒫(𝒜1⊕A2)=𝒫(𝒜1⊕𝒜2)*(∑0≤j≤m(−1)j(mj)(ℬ1⊕ℬ2j(𝒜1⊕𝒜2)m−j)𝒫(𝒜1⊕𝒜2)=𝒫(𝒜1⊕𝒜2)*(∑0≤j≤m(−1)j(mj)(ℬ1j𝒜1m−j⊕ℬ2)j𝒜2m−j))𝒫(𝒜1⊕𝒜2)=𝒫(𝒜1)*(∑0≤j≤m(−1)j(mj)ℬ1j𝒜1m−j)𝒫(𝒜1)⊕𝒫(𝒜2)*(∑0≤j≤m(−1)j(mj)ℬ2j𝒜2m−j)𝒫(𝒜2)=0⊕0=0.

Hence ${𝒜}_{1}⊕{𝒜}_{2}\in [𝒬{HEL}_{\left(m,𝒫\right)}({ℬ}_{1}⊕{ℬ}_{2})]$.

**Proposition 2.16.** *Let* 𝒫∈ℂ[z]⧵{0} *and let* 𝒜,ℬ,𝒱∈ℒ(𝒦)*. If* $𝒜\in [{QHEL}_{\left(m,𝒫\right)}(ℬ)]$ *and* 𝒱 *is an isometry,then* $𝒱𝒜{𝒱}^{*}\in [{QHEL}_{\left(m,𝒫\right)}(𝒱ℬ{𝒱}^{*})]$*.*

*Proof.* Let $P=\sum_{\begin{array}{c} 0\leq k\leq n \end{array}}a_{k}x^{k}$ and ${𝒱}^{*}𝒱=ℐ$ we have


$$\begin{array}{cc} P(𝒱𝒜{𝒱}^{*}) & =\sum_{\begin{array}{c} 0\leq k\leq n \end{array}}a_{k}(𝒱𝒜{𝒱}^{*}{)}^{k}\\ & =\sum_{\begin{array}{c} 0\leq k\leq n \end{array}}a_{k}(𝒱{𝒜}^{k}{𝒱}^{*})\\ & =𝒱\sum_{\begin{array}{c} 0\leq k\leq n \end{array}}a_{k}({𝒜}^{k}){𝒱}^{*}\\ & =𝒱P(𝒜){𝒱}^{*}. \end{array}$$

Hence,


P(𝒱𝒜𝒱*)*Φ(𝒱ℬ𝒱*;𝒱𝒜𝒱*)mP(VA𝒱*)=P(𝒱𝒜𝒱*)*(∑0≤j≤m(−1)j(mj)(𝒱ℬ𝒱*)j(𝒱𝒜𝒱*)m−j)P(𝒱𝒜𝒱*)=(𝒱P(𝒜)𝒱*)*𝒱(∑0≤j≤m(−1)j(mj)(ℬ)j(𝒜)m−j)𝒱*(𝒱𝒫(𝒜)𝒱*)=𝒱P(𝒜)*Φ(ℬ;𝒜)m(ℐ)P(𝒜)𝒱*=0.

Therefore, $𝒱𝒜{𝒱}^{*}\in [{QHEL}_{\left(m,𝒫\right)}(𝒱ℬ{𝒱}^{*}].$ □

Our approach to proving the next lemma follows ideas introduced in [28].

**Lemma 2.17.** *Let* 𝒜,ℬ∈ℒ(𝒦)*.Then the following identity holds for* k∈ℕ*:*


$$\begin{array}{c} \Phi (ℬ;𝒜{)}^{k+1}(ℐ)=ℬ\Phi (ℬ;𝒜{)}^{k}(ℐ)-\Phi (ℬ;𝒜{)}^{k}(ℐ)𝒜. \end{array}$$ (21)

*Proof.* Indeed, we have the following relation:


ℬΦ(ℬ;𝒜)k(ℐ)−Φ(ℬ;𝒜)k(ℐ)𝒜=∑0≤j≤k(−1)k−j(kj)ℬj+1𝒜k−j−∑0≤j≤k(−1)k−j(kj)ℬj𝒜k+1−j=ℬk+1+∑1≤j≤k(−1)k+1−j[(kj−1)+(kj)]ℬj𝒜k+1−j+(−1)k+1𝒜k+1=ℬk+1+∑1≤j≤k(−1)k+1−j(k+1j)ℬj𝒜k+1−j+(−1)k+1𝒜k+1=∑0≤j≤k+1(−1)k+1−j(k+1j)ℬj𝒜k+1−j=Φ(ℬ;𝒜)k+1(ℐ).

**Proposition 2.18.** *Let* 𝒫∈ℂ[z]⧵{0} *and let* $𝒜\in [𝒬{HEL}_{\left(m,𝒫\right)}(ℬ)]$ *for some positive integer m. If* $[{𝒜}^{*},ℬ]=0$*, then* $𝒜\in [𝒬{HEL}_{\left(m+1,𝒫\right)}(ℬ)]$.

*Proof.* Since $𝒜\in [𝒬{HEL}_{\left(m,𝒫\right)}(ℬ)]$, it follows that


$$\begin{array}{c} 𝒫(𝒜{)}^{*}\Phi (ℬ;𝒜{)}^{m}(ℐ)𝒫(𝒜)=0. \end{array}$$

Moreover, according to (21) we have


$$\begin{array}{c} \Phi (ℬ:𝒜{)}^{m+1}(ℐ)=ℬ\Phi (ℬ;𝒜{)}^{m}(I)-\Phi (ℬ;𝒜{)}^{m}(ℐ)(𝒜). \end{array}$$

and we get


$$\begin{array}{cc} 𝒫(𝒜{)}^{*}\Phi (ℬ;𝒜{)}^{m+1}(ℐ)𝒫(𝒜) & =𝒫(𝒜{)}^{*}(ℬ\Phi (ℬ;𝒜{)}^{m}(ℐ)-\Phi (ℬ;𝒜{)}^{m}(ℐ)(𝒜))𝒫(𝒜)\\ & =ℬ𝒫(A{)}^{*}\Phi (B;A{)}^{m}(ℐ)𝒫(𝒜)-𝒫(𝒜{)}^{*}\Phi (ℬ;𝒜{)}^{m}(ℐ)𝒫(𝒜)(𝒜)\\ & =0. \end{array}$$

Hence, $𝒫(𝒜{)}^{*}\Phi (ℬ;𝒜{)}^{m+1}(ℐ)𝒫(𝒜)=0$ and therefore, $𝒜\in [𝒬{HEL}_{\left(m,𝒫\right)}(ℬ)]$.

**Theorem 2.19.** *Let* 𝒫∈ℂ[z]⧵{0} *and let* 𝒜,ℬ∈ℒ(𝒦). *If* $\left({𝒜}_{k}{)}_{k}\in [{QHEL}_{\left(m,𝒫\right)}(ℬ)\right]$ *such that* $\lim_{k\to \infty }\|{𝒜}_{k}-𝒜\|=0,$ *then* $𝒜\in [{QHEL}_{\left(m,𝒫\right)}(ℬ)].$

*Proof.* Fix a nonzero polynomial P∈C[x] and set, for any operator T∈B(K),


M(T):=Φ(B;T)m(I)=∑j=0m(−1)j(mj)BjTm−j.

By hypothesis there is a sequence $\left(A_{k}{)}_{k\geq 1}\subset [{QHEL}_{\left(m,𝒫\right)}(ℬ)\right]$ with $\|A_{k}-A\|\to 0$ as k→∞, and for each *k* we have the identity


$$\begin{array}{c} P(A_{k}{)}^{*}\;M(A_{k})\;P(A_{k})=0. \end{array}$$ (22)

We will pass to the limit in (22). To do this we use two elementary continuity facts about polynomials of operators:

1. For every nonnegative integer *n* the map $T\mapsto T^{n}$ is continuous in operator norm on B(K). Thus $A_{k}^{\;n}\to A^{\;n}$ in norm for every n≤m.
2. If $Q(x)=\sum_{r=0}^{d}c_{r}x^{r}$ is any fixed polynomial then $Q(A_{k})\to Q(A)$ in operator norm (a direct consequence of (1)).

From (2) applied to the polynomial P we obtain


$$\begin{array}{c} \|P(A_{k})-P(A)\|\to 0,\;\|P(A_{k}{)}^{*}-P(A{)}^{*}\|\to 0. \end{array}$$

From (1) applied to the constituent monomials of M(·) we get


‖M(Ak)−M(A)‖=|∑j=0m(−1)j(mj)Bj(Akm−j−Am−j)|→0.

Now estimate the difference


$$\begin{array}{c} \begin{array}{cc} & \left|P(A{)}^{*}\;M(A)\;P(A)-P(A_{k}{)}^{*}\;M(A_{k})\;P(A_{k})\right|\\ & \;\leq |P(A{)}^{*}|\;|M(A)-M(A_{k})|\;|P(A)|\\ & \;+|P(A{)}^{*}-P(A_{k}{)}^{*}|\;|M(A_{k})|\;|P(A)|\\ & \;+|P(A_{k}{)}^{*}|\;|M(A_{k})|\;|P(A)-P(A_{k})|. \end{array} \end{array}$$

Each term on the right tends to 0 as k→∞, because the factors $P(A_{k}{)}^{\left(*\right)}\to P(A{)}^{\left(*\right)}$ and $M(A_{k})\to M(A)$ in operator norm, and the norms $\left\|P(A_{k})\|,\|M(A_{k})\right\|$ remain uniformly bounded for large *k*. Therefore


$$\begin{array}{c} \lim_{k\to \infty }|P(A{)}^{*}M(A)P(A)-P(A_{k}{)}^{*}M(A_{k})P(A_{k})|=0. \end{array}$$

But each term $P(A_{k}{)}^{*}M(A_{k})P(A_{k})$ equals 0 by (1), so the limit above is simply


$$\begin{array}{c} \|P(A{)}^{*}M(A)P(A)\|=0. \end{array}$$

Hence


$$\begin{array}{c} P(A{)}^{*}\;M(A)\;P(A)=0, \end{array}$$

which shows $A\in [{QHEL}_{\left(m,P\right)}(B)]$. This completes the proof.

**Theorem 2.20.** *Let* 𝒫∈ℂ[z]⧵{0} *and let* 𝒜,ℬ∈ℒ(𝒦)*. Assume that* 𝒜 *belongs to the polynomially quasi strict Hilton class of* ℬ *with order m. If the commutator* $[{𝒜}^{*},ℬ]=0$*, then the family of linear transformations*


$$\begin{array}{c} \left\{𝒫(𝒜{)}^{*}\Phi (ℬ,𝒜{)}^{j}(ℐ)𝒫(𝒜)\;|\;j=0,1,...,m-1\right\} \end{array}$$

*is linearly independent.*

*Proof.* In view of (21), we have


$$\begin{array}{c} \Phi (ℬ;𝒜{)}^{k}(ℐ)=ℬ\Phi (ℬ;𝒜{)}^{k-1}(ℐ)-\Phi (ℬ;𝒜{)}^{k-1}(ℐ)𝒜\;\text{for all }k\geq 1. \end{array}$$

Now, we compute:


$$\begin{array}{c} 𝒫(𝒜{)}^{*}\Phi (ℬ,𝒜)(ℐ{)}^{k}𝒫(𝒜)={𝒫}^{*}(𝒜)\left(B\Phi_{k-1}(ℬ,𝒜{)}^{k-1}(ℐ)-\Phi (ℬ,𝒜{)}^{k-1}(ℐ))𝒜\right)𝒫(𝒜). \end{array}$$

Since ${𝒜}^{*}$ and ℬ commute (i.e., $[{𝒜}^{*},ℬ]=0$), this simplifies to:


$$\begin{array}{c} 𝒫(𝒜{)}^{*}\Phi (ℬ,𝒜)(ℐ{)}^{k}𝒫(𝒜)=ℬ𝒫(𝒜)\Phi (ℬ,𝒜{)}^{k-1}(ℐ)𝒫(A)-𝒫(𝒜)\Phi (ℬ;𝒜{)}^{k-1}(ℐ)𝒫(𝒜)𝒜. \end{array}$$

Now assume that for some complex numbers $\mu_{j}$, we have


$$\begin{array}{c} \sum_{0\leq k\leq m-1}\mu_{k}𝒫(𝒜{)}^{*}\Phi (ℬ;𝒜{)}^{k}(ℐ)𝒫(𝒜)=0. \end{array}$$ (23)

Multiplying equation (23) on the left by ℬ, and using the fact that $[{𝒜}^{*},ℬ]=0$, we obtain:


$$\begin{array}{c} \sum_{0\leq k\leq m-1}\;\mu_{k}𝒫(𝒜{)}^{*}ℬ\Phi (ℬ,𝒜{)}^{k}(ℐ)𝒫(𝒜)=0. \end{array}$$ (24)

Similarly, multiplying equation (23) on the right by 𝒜, we get:


$$\begin{array}{c} \sum_{0\leq k\leq m-1}\mu_{k}𝒫(𝒜{)}^{*}\Phi (ℬ,𝒜{)}^{k}(ℐ)𝒫(𝒜)𝒜=0. \end{array}$$ (25)

Subtracting equation (25) from (24), we obtain:


$$\begin{array}{c} \sum_{0\leq m-1}\mu_{k}𝒫(𝒜{)}^{*}\left(ℬ\Phi (ℬ;𝒜{)}^{k}(ℐ)-\Phi (ℬ;𝒜)(ℐ)𝒜\right)𝒫(𝒜)=0. \end{array}$$ (26)

By (21), the expression inside the parentheses is $\Phi (ℬ,𝒜{)}^{k+1}(ℐ)$, so equation (26) becomes:


$$\begin{array}{c} \sum_{0\leq \leq m-1}\mu_{k}𝒫(𝒜{)}^{*}\Phi (ℬ,𝒜)(I{)}^{k+1}𝒫(𝒜)=0. \end{array}$$ (27)

Applying the same procedure repeatedly to equation (27), we obtain:


$$\begin{array}{c} \sum_{0\leq k\leq m-1}\mu_{k}𝒫(𝒜{)}^{*}\Phi (ℬ,𝒜{)}^{k+2}(ℐ)𝒫(𝒜)=0, \end{array}$$ (28)

and continuing this process yields:


$$\begin{array}{c} \sum_{0\leq k\leq m-1}\mu_{k}𝒫(𝒜{)}^{*}\Phi (ℬ;𝒜{)}^{k+r}(ℐ)𝒫(𝒜)=0\;\text{for all }r\in \mathbb{N}. \end{array}$$ (29)

From Proposition 2.18, it is known that if $𝒜\in [𝒬{HEL}_{(m,P)}(ℬ)]$, then


$$\begin{array}{c} 𝒜\in [𝒬{HEL}_{\left(q,𝒫\right)}(ℬ)]\;\text{for all }q\geq m. \end{array}$$

This implies the following: For r=m−1, equation (29) becomes


$$\begin{array}{c} \sum_{0\leq k\leq m-1}\mu_{k}𝒫(𝒜{)}^{*}\Phi (ℬ,𝒜{)}^{k+m-1}(ℐ)𝒫(𝒜)=0. \end{array}$$

In particular, this implies:


$$\begin{array}{c} \mu_{0}𝒫(𝒜{)}^{*}\Phi (ℬ,𝒜{)}^{m-1}(ℐ)𝒫(𝒜)=0. \end{array}$$

But since $𝒫(𝒜{)}^{*}\Phi (ℬ,𝒜{)}^{m-1}(ℐ)𝒫(𝒜)\text{≠}0$, it follows that $\mu_{0}=0$.

Similarly, for r=m−2, we obtain:


$$\begin{array}{c} \mu_{1}𝒫(𝒜{)}^{*}\Phi (ℬ,A{)}^{m-1}(ℐ)𝒫(𝒜)=0\Rightarrow \mu_{1}=0, \end{array}$$

and repeating this process for r=m−3,...,1,0, we conclude that


$$\begin{array}{c} \mu_{k}=0\;\text{for all }k=0,1,...,m-1. \end{array}$$

Hence,


$$\begin{array}{c} \sum_{0\leq k\leq m-1}\mu_{k}𝒫(𝒜{)}^{*}\Phi (ℬ,𝒜{)}^{k}(ℐ)𝒫(𝒜)=0\;\Rightarrow \;\mu_{0}=\mu_{1}=...=\mu_{m-1}=0. \end{array}$$

This proves that the set $\{𝒫(𝒜{)}^{*}\Phi (ℬ;𝒜{)}^{k}(ℐ)𝒫(𝒜){\}}_{k=0}^{m-1}$ is linearly independent.

In the sequel, we establish several propositions that characterize the approximate spectrum and the point spectrum of the operators 𝒜 and ℬ satisfying $𝒜\in [{QHEL}_{\left(m,𝒫\right)}(ℬ)]$.

**Proposition 2.21.** *Let* P∈C[x]⧵{0} *and suppose* $A\in 𝒬{HEL}_{\left(m,P\right)}(B)$*. If* $\lambda \in \sigma_{p}(A)$ *and* P(λ)≠0*, then*


$$\begin{array}{c} \Phi (B;\lambda {)}^{m}(I)\;x\in \ker(A-\lambda I{)}^{⟂}. \end{array}$$

*Proof.* Let $\lambda \in \sigma_{p}(A)$ and let x≠0 satisfy (A−λI)x=0. By hypothesis P(λ)≠0 and P(A)x=P(λ)x. The defining identity for A is


$$\begin{array}{c} P(A{)}^{*}\;\Phi (B;A{)}^{m}\;P(A)=0, \end{array}$$

where we write Φ(B;A)m=∑j=0m(−1)j(mj)BjAm−j. Apply this identity to the vector *x* to obtain


0=P(A)*(∑j=0m(−1)j(mj)BjAm−j)P(A)x.

Using $A^{\;m-j}x=\lambda^{\;m-j}x$ and P(A)x=P(λ)x we get


0=P(A)*(∑j=0m(−1)j(mj)Bjλm−j)P(λ)x=P(λ)P(A)*Φ(B;λ)m(I)x.

Since P(λ)≠0 we conclude


$$\begin{array}{c} P(A{)}^{*}\;\Phi (B;\lambda {)}^{m}(I)\;x=0. \end{array}$$

Now let y∈ker(A−λI). Then P(A)y=P(λ)y, and for the inner product


$$\begin{array}{c} 0=\langle P(A{)}^{*}\;\Phi (B;\lambda {)}^{m}(I)\;x,\;y\rangle =\langle \Phi (B;\lambda {)}^{m}(I)\;x,\;P(A)y\rangle =\overset{―}{P(\lambda )}\langle \Phi (B;\lambda {)}^{m}(I)\;x,\;y\rangle . \end{array}$$

Because P(λ)≠0 we obtain $\langle \Phi (B;\lambda {)}^{m}(I)\;x,\;y\rangle =0$ for every y∈ker(A−λI). Hence


$$\begin{array}{c} \Phi (B;\lambda {)}^{m}(I)\;x\in \ker(A-\lambda I{)}^{⟂}, \end{array}$$

as required.

**Proposition 2.22.** *Let* P∈C[x]⧵{0} *and* $A\in 𝒬{HEL}_{\left(m,P\right)}(B)$*. If* $\lambda \in \sigma_{ap}(A)$ *and* P(λ)≠0*, then there exists a sequence* $(x_{n})\subset H$*,* $\|x_{n}\|=1$*, such that*


$$\begin{array}{c} P(A{)}^{*}\;\Phi (B;\lambda {)}^{m}(I)\;x_{n}\to 0. \end{array}$$

*The images of* $\Phi (B;\lambda {)}^{m}(I)$ *are asymptotically orthogonal to the range of* P(A),*, i.e., for the approximate eigenvector sequence* $\left(x_{n}\right)$, $\langle \Phi (B;\lambda {)}^{m}(I)\;x_{n},P(A)\;y\rangle ⟶0\;\text{for all }y\in H.$

*Proof.* Since $\lambda \in \sigma_{ap}(A)$, by definition there exists a sequence $(x_{n})\subset H$ with $\|x_{n}\|=1$ such that


$$\begin{array}{c} (A-\lambda I)x_{n}\to 0\;\text{as }n\to \infty . \end{array}$$

The defining identity of a $Q{HEL}_{\left(m,P\right)}$-operator is


$$\begin{array}{c} P(A{)}^{*}\;\Phi (B;A{)}^{m}\;P(A)=0, \end{array}$$

where


Φ(B;A)m=∑j=0m(−1)j(mj)BjAm−j.

Applying this identity to the approximate eigenvector $x_{n}$ gives


$$\begin{array}{c} P(A{)}^{*}\;\Phi (B;A{)}^{m}\;P(A)x_{n}=0. \end{array}$$

Since $(A-\lambda I)x_{n}\to 0$ and P is continuous as a polynomial, we have


$$\begin{array}{c} A^{\;m-j}x_{n}\approx \lambda^{\;m-j}x_{n},\;P(A)x_{n}\approx P(\lambda )x_{n} \end{array}$$

for large *n*. Substituting into the identity, we obtain


$$\begin{array}{c} P(A{)}^{*}\;\Phi (B;\lambda {)}^{m}(I)\;P(\lambda )x_{n}\to 0. \end{array}$$

Because P(λ)≠0, dividing by this scalar yields


$$\begin{array}{c} P(A{)}^{*}\;\Phi (B;\lambda {)}^{m}(I)\;x_{n}\to 0. \end{array}$$

Finally, for any y∈H, we have


$$\begin{array}{c} \langle \Phi (B;\lambda {)}^{m}(I)\;x_{n},P(A)\;y\rangle =\langle P(A{)}^{*}\;\Phi (B;\lambda {)}^{m}(I)\;x_{n},y\rangle \to 0, \end{array}$$

so the images of $\Phi (B;\lambda {)}^{m}(I)$ are asymptotically orthogonal to the range of P(A) along the sequence $\left(x_{n}\right)$.

## 3. Conclusion

In this work, we introduced and systematically studied the class of *polynomially-quasi Helton operators* $\left[{QHEL}_{\left(m,P\right)}(ℬ)\right]$, which generalizes the classical Helton operators by incorporating a polynomial quasi-condition. We provided rigorous definitions, explored fundamental properties, and established key structural results, including spectral behavior, stability under limits, and the behavior under invariant subspaces. These results demonstrate that the PQ-Helton class forms a robust and well-structured extension of the Helton operator framework. This study provides a comprehensive framework for understanding polynomially-quasi Helton operators, bridges a gap between abstract operator-theoretic concepts and concrete examples, and opens several avenues for further research in infinite-dimensional operator theory.
